# Deployment and use of mobile phone technology for real-time reporting of fever cases and malaria treatment failure in areas of declining malaria transmission in Muheza district north-eastern Tanzania

**DOI:** 10.1186/s12936-017-1956-z

**Published:** 2017-08-01

**Authors:** Filbert Francis, Deus S. Ishengoma, Bruno P. Mmbando, Acleus S. M. Rutta, Mwelecele N. Malecela, Benjamin Mayala, Martha M. Lemnge, Edwin Michael

**Affiliations:** 1 National Institute for Medical Research, Tanga Research Centre, Tanga, Tanzania; 2 National Institute for Medical Research, Headquarters, Dar es Salaam, Tanzania; 3 University of Notre Dame, South Bend, IN USA

**Keywords:** Malaria, Anti-malarials, RDTs, Artemether–lumefantrine, Drug resistance, Mobile phone application, Village health workers

## Abstract

**Background:**

Early detection of febrile illnesses at community level is essential for improved malaria case management and control. Currently, mobile phone-based technology has been commonly used to collect and transfer health information and services in different settings. This study assessed the applicability of mobile phone-based technology in real-time reporting of fever cases and management of malaria by village health workers (VHWs) in north-eastern Tanzania.

**Methods:**

The community mobile phone-based disease surveillance and treatment for malaria (ComDSTM) platform, combined with mobile phones and web applications, was developed and implemented in three villages and one dispensary in Muheza district from November 2013 to October 2014. A baseline census was conducted in May 2013. The data were uploaded on a web-based database and updated during follow-up home visits by VHWs. Active and passive case detection (ACD, PCD) of febrile cases were done by VHWs and cases found positive by malaria rapid diagnostic test (RDT) were given the first dose of artemether–lumefantrine (AL) at the dispensary. Each patient was visited at home by VHWs daily for the first 3 days to supervise intake of anti-malarial and on day 7 to monitor the recovery process. The data were captured and transmitted to the database using mobile phones.

**Results:**

The baseline population in the three villages was 2934 in 678 households. A total of 1907 febrile cases were recorded by VHWs and 1828 (95.9%) were captured using mobile phones. At the dispensary, 1778 (93.2%) febrile cases were registered and of these, 84.2% were captured through PCD. Positivity rates were 48.2 and 45.8% by RDT and microscopy, respectively. Nine cases had treatment failure reported on day 7 post-treatment and adherence to treatment was 98%. One patient with severe febrile illness was referred to Muheza district hospital.

**Conclusion:**

The study showed that mobile phone-based technology can be successfully used by VHWs in surveillance and timely reporting of fever episodes and monitoring of treatment failure in remote areas. Further optimization and scaling-up will be required to utilize the tools for improved malaria case management and drug resistance surveillance.

## Background

Malaria is a disease of global health concern that compromises the growth and stability of low- and middle-income countries (LMICs) in which it is endemic [1]. Malaria control largely depends on case management with effective anti-malarials together with insecticide-treated/long-lasting bed nets (ITNs/LLINs) and indoor residual spraying (IRS) [2]. However, current therapy is losing effectiveness because malaria pathogens are increasingly becoming tolerant/resistant to drugs [3, 4] and other vector control strategies are compromised by the rapidly emerging insecticide resistance among mosquito populations [5]. For patients developing severe disease, early detection is important for effective treatments [6]. Monitoring of drug resistance and severe disease are major challenges and yet critical to sustaining the public health gains made in those areas where malaria transmission has been substantially reduced by enhanced investment and deployment of different interventions.

Recent epidemiological studies have shown that despite a decline in the burden of malaria and some bacterial infections in areas, which were halo/hyper-endemic to the disease, febrile infections have remained unchanged [6, 7]. Detection and management of both malaria and non-malaria febrile illnesses need to be improved and intensified if communities are to benefit from the declining trends of the malaria burden. Furthermore, detecting pathogens that are drug-resistant or associated with severe disease in real-time as they emerge in the population and transferring them to a laboratory setting is essential for proper patient care, informing policy and developing new drugs that combat resistance. Detecting severe disease in early stages is expected to improve therapeutic options and dramatically reduce mortality.

A 2009 white paper authored by Vital Wave Consulting and sponsored by the UN Foundation–Vodafone Foundation Partnership described 51 different mobile health projects that range from education and awareness to data collection and monitoring, and epidemic tracking. Use of mobile phones is higher than any other technology in the developing world [8]. LMICs are beginning to deploy mobile phone-based tools that facilitate collection and reach of health information and services to remote populations via trained community health workers (CHWs) [9]. This promotes a shift toward community care and effective health promotion. However monitoring and detecting the emergence of drug resistance and severe disease at the population level and pipelining them to the appropriate laboratory and/or clinical settings for analysis and care, respectively, is lacking.

Development of resistance to anti-malarial drugs and severe disease in malaria are rare events and are difficult and costly to capture using current passive or active surveillance methods. It will be the case particularly in areas where malaria is declining under the pressure of interventions. Early detection of recurrent fever cases under such a circumstance will provide a timely warning that the pathogen may not be responding to interventions, including treatment, meaning that case management strategies may require change to contain and reduce such evolving responses. The increasing deployment of mobile technology to allow tracking of patient health data at the point of service using CHWs will facilitate a reliable cost-effective tool for routine real-time detection of these events from the field. Recent experience from Tanzania’s neglected tropical disease control programme, in which CHWs were successfully trained to use mobile phones for capturing and tracking household treatment and clinical data, highlights the applicability of the proposed tool in the Tanzanian settings.

The overall goal of this pilot study was to develop, test and deploy a mobile phone-based platform and assess its potential use in surveillance of febrile illnesses, including severe cases, supporting prompt malaria case management and monitoring of treatment outcomes in areas of changing malaria epidemiology. The community-based disease surveillance and treatment of malaria (ComDSTM) system was developed, optimized and effectively used in an area where malaria has progressively declined in recent years. The findings are discussed in the perspective of potential application of the ComDSTM system in areas under transition to pre-elimination of malaria in sub-Saharan Africa.

## Methods

### Study site

**Fig. 1:**
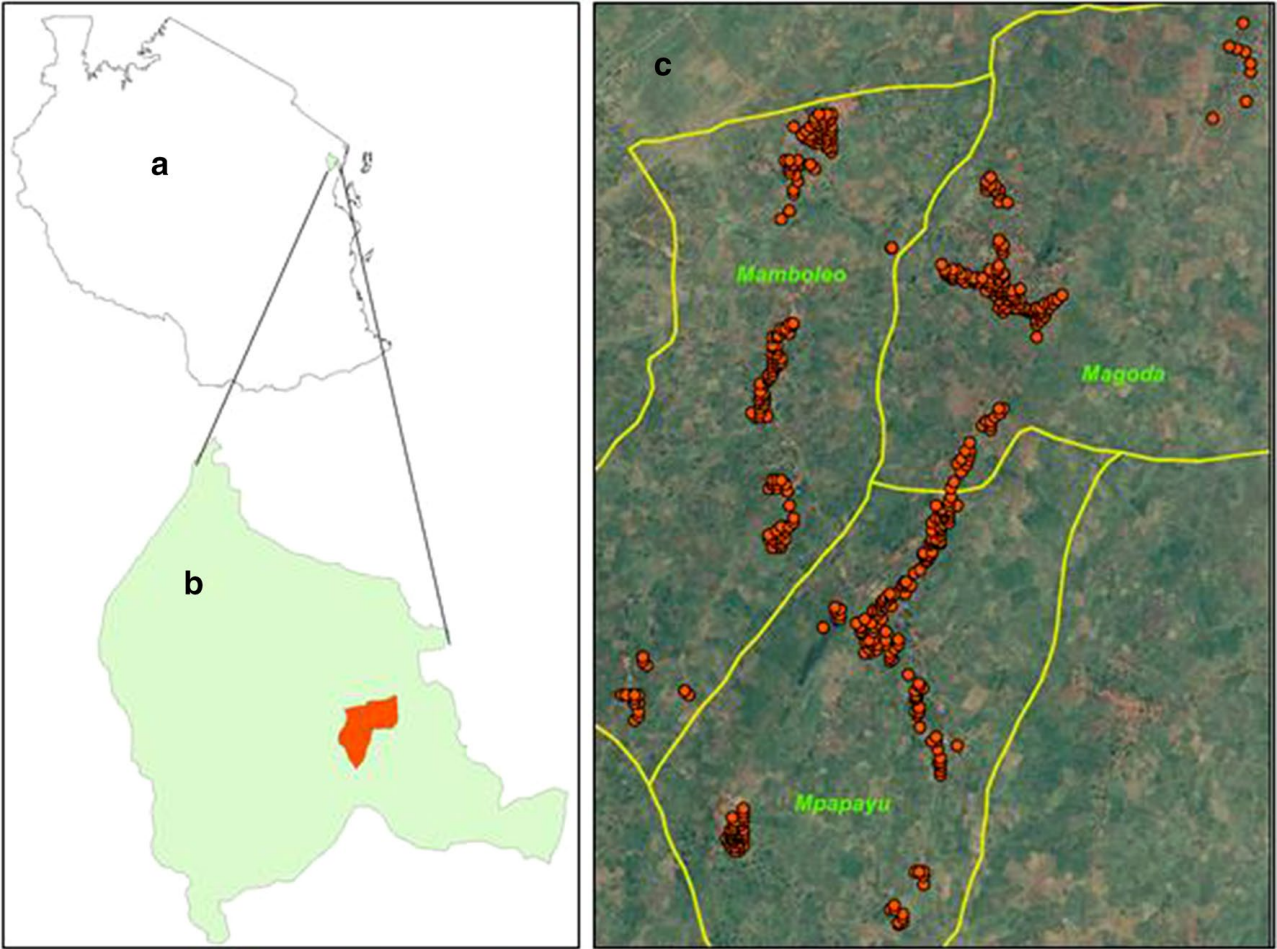
Map of Tanzania (**a**) showing the study villages (**b**) and the household locations (**c**)

The ComDSTM system was implemented in three villages of Mamboleo, Magoda and Mpapayu in Muheza district, northeastern Tanzania. The population of these villages was 2594 individuals in 654 households according to the baseline population census conducted in 2010 and the distribution of the households is shown in Fig. 1. Prior to the implementation of the ComDSTM system, the population data was updated in May 2013. The study villages have been involved in previous studies of malaria and lymphatic filariasis since 1992 (Mamboleo and Magoda) and 1997 (Mpapayu) as previously described [10–12].

### Study design

The study was a prospective, longitudinal surveillance using mobile phones to actively and passively monitor the occurrence of febrile illnesses and treatment of malaria by village health workers (VHWs). All individuals from all households in the three villages were targeted.

### Development of ComDSTM system

The ComDSTM mobile phone-based system was conceptualized by a team of researchers at the National Institute for Medical Research (NIMR) in Tanga, in collaboration with the University of Notre Dame, Indiana (USA), and developed by Rain Concert, an IT company from India. The application was developed to run on android mobile phones. The development of the application followed all standard software development life cycle (system design, implementation, testing, and validation) whereby all procedures involved in malaria case management at the community and health facility level (dispensary) was digitized.

**Fig. 2:**
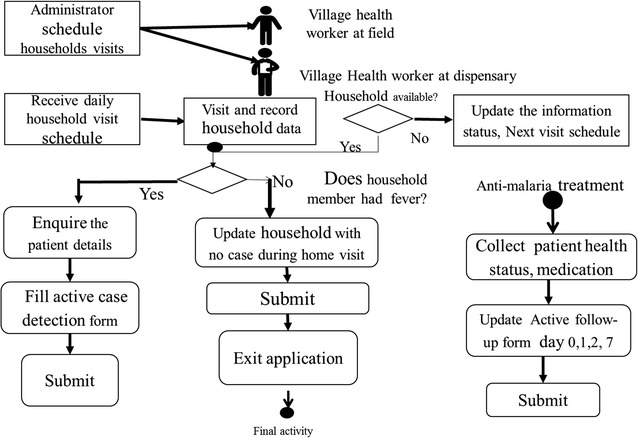
Conceptual framework and information flow between different actors of the ComDSTM

The ComDSTM system was designed to facilitate the detection of fever cases by VHWs at the household/community level through active case detection (ACD) and sending identified cases to the dispensary for appropriate management. The system tracked patients detected at the household as they reported to the dispensary for laboratory diagnosis, sample collection and treatment. Similarly, patients who passively reported to the dispensary were captured via the passive case detection (PCD) component and channelled to the clinician and laboratory for diagnosis, sample collection and treatment. The system also supported follow-up of patients on treatment in order to ensure drugs were taken and to document the treatment outcome on day 7. All the data collected in the community through ACD or follow-up of patients and at the dispensary (PCD, laboratory testing and treatment) were captured using mobile phones and synchronized to a web-based database. The conceptual flow of events and information from and between different actors of the ComDSTM is shown in Fig. 2.

The main components of the ComDSTM included applications for updating household demographic data, registration of new households, collection of socio-economic status of new households, recording of fever cases at the community level (ACD) and registration of all fever patients at the dispensary from both ACD and PCD. Other applications covered clinical assessment of patients by clinician, recording laboratory diagnosis done at the dispensary using malaria rapid diagnostic tests (RDTs) and the test results, treatment of malaria with artemether–lumefantrine (AL), generating dosing schedule of anti-malarials and reporting of other drugs prescribed. A monitoring application was developed to allow VHWs to make follow-up of patients on AL treatment by visiting patients at home to give them the second and subsequent doses of anti-malarial (from day 0 to day 3), to monitor treatment outcome and collect blood samples [blood slides and dried blood spots on filter papers (DBS)] on day 7. Sample tracking applications were used in the laboratory to keep records of blood samples collected from malaria-positive patients on days 0 and 7, as well as details of samples stored at the Amani Biomedical Research Laboratory (AMBRELA), the main laboratory of NIMR in Tanga. The samples stored at AMBRELA were archived with consent from study participants and they will be analysed in the future to determine the genetic/genomic profiles of parasites and blood donors, drug levels and detection of non-malaria fever causing pathogens. Reporting applications were also developed for presentation of the reports in both textual and graphical format. They also provided summary statistics at the individual, household and village levels.

## Data collection procedures

### Collection and updating of demographic data and socio-economic status

**Fig. 3:**
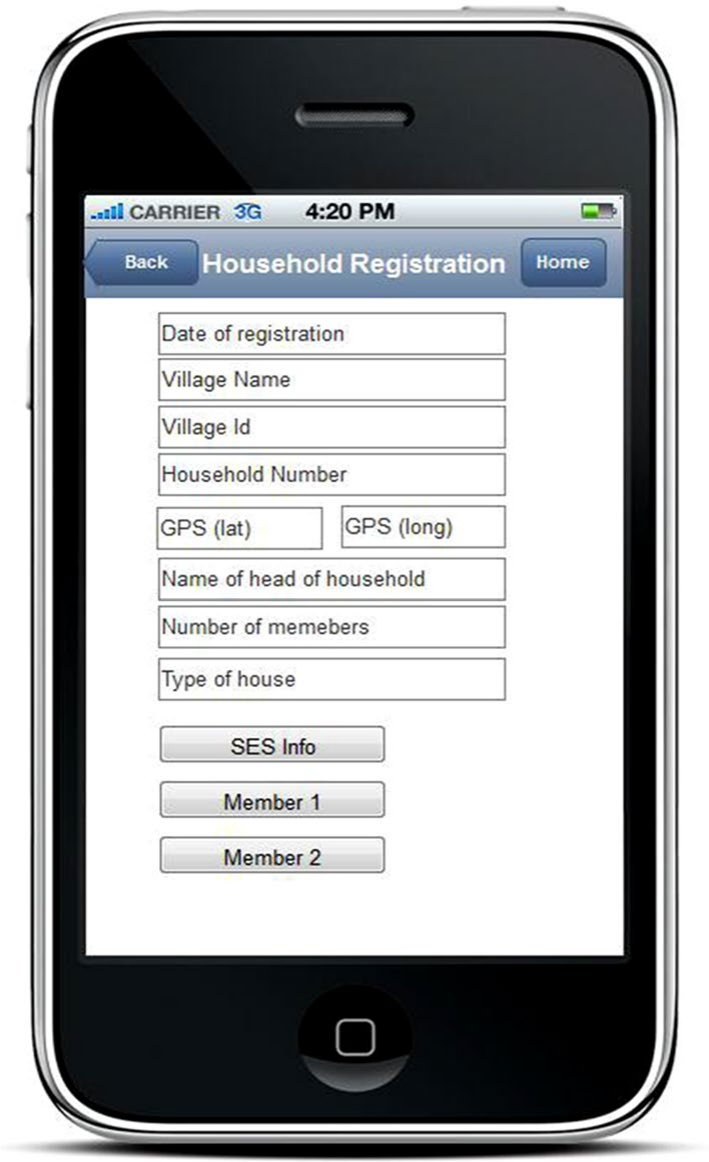
A mobile phone window for registration and updating the household information, members’ registration and assessing household socio-economic status. *Id* identification number, *SES* socio-economic status, *GPS* geographical positioning system, *lat* latitude, *long* longitude

Following the census survey which was conducted in May 2013, updates of household demographic data were routinely done during household visits once weekly by the VHWs from November 2013. Activities involved updates of household records collected during the May 2013 census, registration of new households, registration of vital events (deaths and births), recording in and out-migrations, geographical positions (latitude and longitude) of the households, and registration of new household members. The socio-economic status of each household was assessed during the baseline census and it was only done for the new household registered during follow-up visits by VHWs. All data collected were uploaded in the database using mobile phones applications. During the baseline census, each person was assigned a unique identification number (IDN), which was used later to link and retrieve individual information during follow-up. Similarly, all households were assigned IDN for tracking and updating the household and its members’ records during follow-up (Fig. 3).

### Active and passive detection of fevers

Two VHWs were deployed in each of the three villages for ACD of fevers while two were deployed at Magoda dispensary, which is located at the centre of the three villages, to receive ACD cases identified from the community and febrile patients who passively reported to the dispensary through PCD. The VHWs visited each of the households once weekly to identify any household member with fever at presentation (axillary temperature ≥37.5 °C) or a history of fever within 48 h. All cases identified with fevers through ACD were referred to Magoda dispensary. Patients with recurrent fever after initial treatment were identified during ACD and sent back to the dispensary in case of treatment failure. At Magoda dispensary, patients received from ACD or on a self-initiated visit through PCD were registered using the PCD application to initiate the clinical process. Axillary body temperature and weight were measured and the information was captured on the mobile phone and channelled to the clinician for further diagnosis and management.

### Clinical diagnosis

**Fig. 4:**
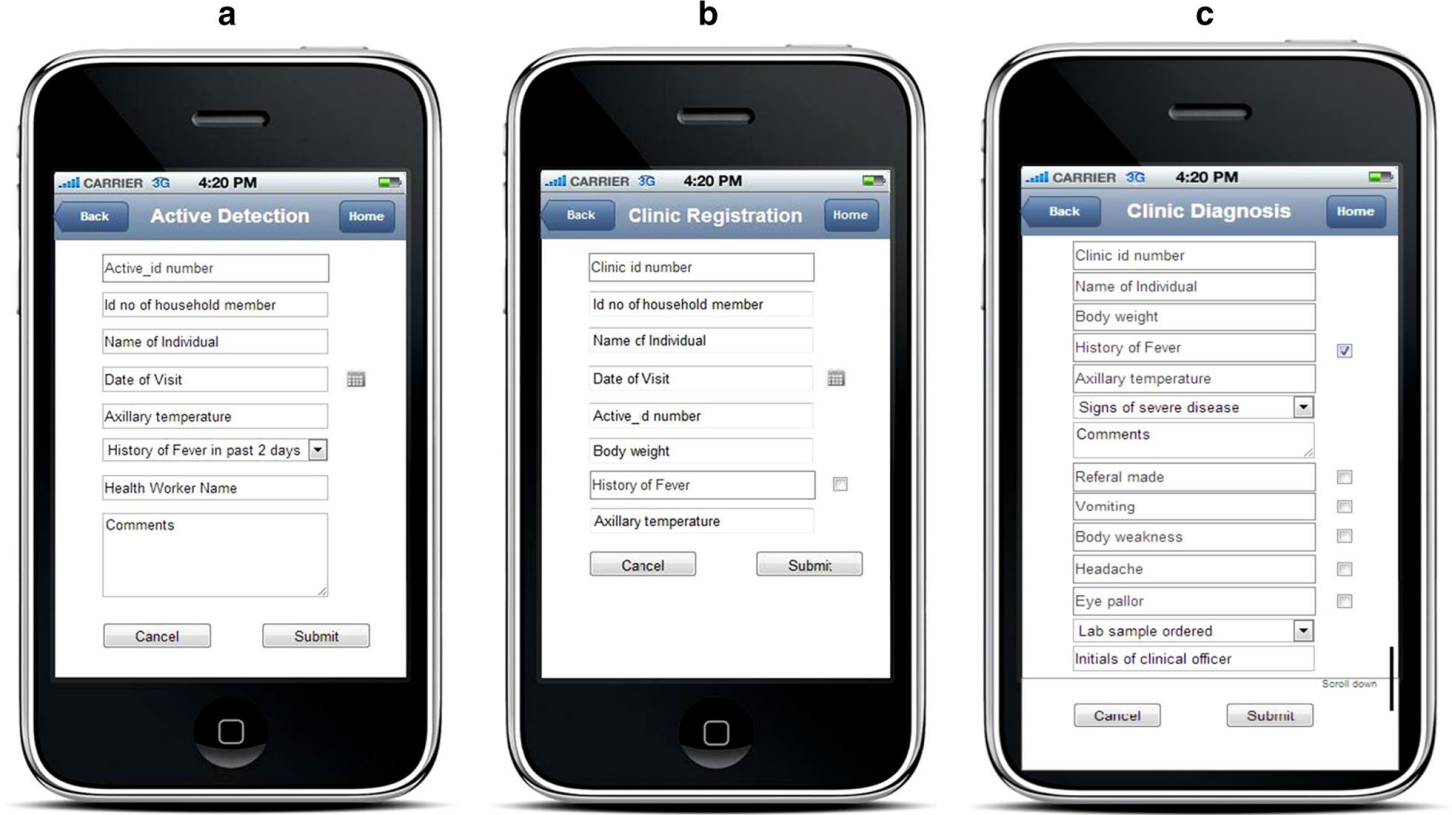
Panel of screen shots of mobile phone application interfaces for collection of active cases, passive cases and clinical diagnosis. **a** Active case collection, **b** clinic registration, **c** clinical diagnosis

At the dispensary, a clinician took further history and carried out a physical examination of patients. A checklist with symptoms of malaria, such as vomiting, body weakness, headache, eye pallor, was prepared in the application and used for initial diagnosis before a patient was sent to the laboratory. The clinician transmitted and shared the information with laboratory staff and then referred the patients to laboratory for malaria testing by RDTs if malaria was suspected. Diagnosis of other causes of fever and their treatment were also explored and recorded. All data were collected using mobile phone applications (Fig. 4).

### Laboratory diagnosis

At the laboratory, a nurse collected a finger prick blood sample for detection of malaria parasites by RDTs, preparation of blood slides for detection of malaria parasites by microscopy and DBS. About 100–300 ml of whole blood was collected in EDTA tubes for analysis of parasites and human genetic factors, and other causes of febrile infections. Confirmatory diagnosis of malaria was made at the dispensary and treatment was administered based on the RDT results. Additionally, blood slides (thick and thin) were prepared for microscopic detection of malaria parasites, which was done later in the laboratory in Tanga. All samples and used RDTs were sent to Tanga on the same day for processing and storage. The results of RDTs and details of the collected samples were recorded and transmitted to the web-based database using mobile phones. The RDTs results were also channelled to the clinician for patients management.

### Final diagnosis, treatment with anti-malarials and follow-up of patients

**Fig. 5:**
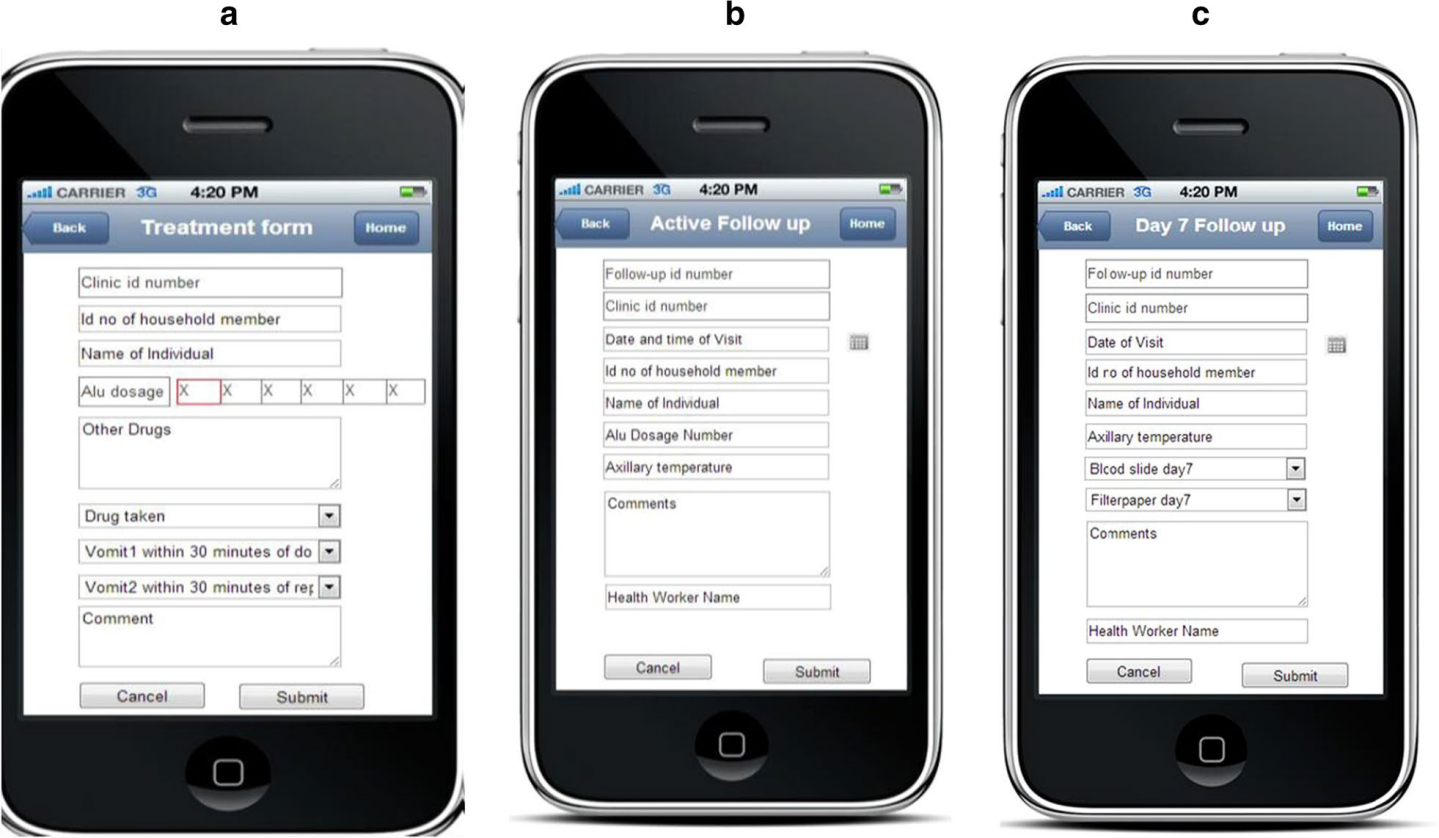
Panel of screen shots of mobile phone application interfaces for capturing information on treatment, active follow-up for intake of anti-malarial and active follow-up at day 7. *Id* identification number, *AL* artemether–lumefantrine; **a** treatment form, **b** active follow-up form, **c** day 7 follow-up

The laboratory results were sent to the clinician and used for final diagnosis of malaria. For patients with positive RDTs results, the clinician prescribed appropriate drugs and sent the information to the project nurse who administered the first dose of AL at the dispensary. The patient was observed for 30 min to ensure the drugs were not vomited. If the patients vomited within 30 min, a full dose was repeated and recorded using a treatment application form on the mobile (Fig. 5). Information of treatment was shared with the respective VHWs located in the patient’s village through mobile phones. A visit schedule was generated and sent to the VHWs to facilitate home visits to administer subsequent doses of AL. The AL doses were generated for four categories: one tablet for children weighing 5–14.9 kg; two tablets for children between 15 and 24.9 kg; three tablets for those 25–34.9 kg; four tablets for adults weighing ≥35 kg. The drugs were taken twice a day for 3 days, 8 h apart on the 1st day and 12-h for the remaining 2 days. All doses of AL were taken under the supervision of either the nurse (at the dispensary) or the VHWs (during home visits). The VHWs visited the patient on day 7 post-treatment to assess the cure rate and take blood samples for future assay of plasma lumefantrine (Fig. 5). Patients who were detected to have malaria parasites on day 7 post treatment were re-treated using AL.

## Reports

An application was developed for generating reports in both graphical and textual formats in real time and cumulatively. The reports included summary statistics of the cases attended at the individuals and household levels, lists of symptoms associated with malaria, including vomiting, headache and body pain, a summary of treatment outcomes and the list of individuals with recurrent episodes of fevers. It also provided the types and frequencies of patients presenting with malaria symptoms as assessed by the clinician and spatial distribution of cases in the study villages linked with Google maps.

### Central administration of the system

The system administrator was responsible for ensuring that ComDSTM was operating optimally. This included installation of applications on all mobile phones and setting up internet connectivity in each phone. The administrator scheduled and allocated daily tasks to each VHW, validated the data transmitted to the server and resolved queries raised by all users of the ComDSTM. Data were transferred between applications remotely in both textual and graphical format. Summary statistics at household and individual level were generated, including demographic characteristics of each recurrent fever and severe disease cases, clinical characteristics and risk factors associated with recurrent fevers and malaria.

### Database implementation

The database was implemented using MYSQL and APACHE. A web application was implemented using PHP and the mobile applications were implemented using Android operating system. The domain name was created and registered to enhance access speed of the different applications running on the android-based mobile phones.

### Training of village health workers and other system users

Training of all project staff was conducted for 5 days in October 2013. The initial training involved a brief overview of the study protocol, introduction to standard operating procedures and the framework and modules of ComDSTM. Thereafter, an intensive training was provided in the field for an additional 7 days before actual data collection. In the field, the data collection team including VHWs and dispensary staff (nurses and a clinical officer) was given an opportunity to study and familiarize themselves with all components of the ComDSTM. They were trained on how to troubleshoot in case they experienced problems with the system. The nurses and a clinician were trained on how to carry out a physical examination, perform RDT, collect blood samples, and prepare blood slides and label samples. Laboratory personnel at AMBRELA were trained on how to use the web applications for posting and retrieving laboratory results as well as sample storage and archiving. Weekly supervision was undertaken by three members of the technical team composed of a laboratory technician, database expert and a researcher/clinician to provide technical support to the data collection team and maintain the quality of the work.

### Data management and analysis

The data generated were managed directly through the web-based database. As part of quality control, the data were also checked daily and weekly through the web applications. The process of checking data included correcting errors and resolving issues raised by the data collection team (VHWs and staff at the dispensary). Some of the changes implemented included deleting duplicated records. The system administrator supervised data entry for all cases recorded on paper when the system was offline because of problems with internet connectivity. All data stored in the web-based database were exported to Excel and STATA version 11 (Stata Corp, College Station, USA) for analysis. Descriptive statistics were used to generate summary statistics of different variables, and the results were presented using frequencies and proportions. Chi square test was used to compare categorical variables whereas Student *t* test or ANOVA were used to compare continuous variables (e.g., age and parasite density) between groups. p value <0.05 was considered to be significant.

## Results

### Demographic characteristics of study population

During the baseline census, which was conducted in May 2013, the population in the three villages was 2934 in 678 households with a median age of 19.1 years (interquartile range (IQR) = 18.1–40.1 years) (Table 1). The majority of the residents (55.2%) were of the Bondei tribe and 50.4% of all residents were aged ≥20 years. The mean household size was four people (range 1–19) and there was no significant difference among the study villages (p = 0.09). Overall, 73% of the households (n = 615) owned at least one bed net while 49.7% of the households owned two to three bed nets. Most of the bed nets were either treated with insecticides or were LLINs (Table 1).

**Table 1:** Demographic characteristics of the study population at baseline

Variable	Number (%)
Population at baseline	2934
Gender	
Female	1522 (51.9)
Male	1412 (48.1)
Age–median (IQR)	19.1 (8.1–40.1)
Tribe	
Bondei	1620 (55.2)
Sambaa	458 (15.6)
Zigua	145 (4.9)
Mmakonde	109 (3.7)
Muha	49 (1.7)
Others	553 (23.2)
Age groups in years	
<1	60 (2.0)
1–4	315 (10.7)
5–9	420 (14.3)
10–14	383 (13.1)
15–19	276 (9.4)
20+	1480 (50.4)
Owned bed net^a^	
Not owned any bed net	162 (26.3)
Owned at least 1 bed net	453 (73.7)
Number of bed nets per household^a^	
1	199 (43.9)
2–3	225 (49.7)
4–5	22 (4.5)
5–12	7 (1.2)

^a^Number of households which responded = 615/678 (91.0%)

### Detection and reporting of febrile and malaria cases

**Fig. 6:**
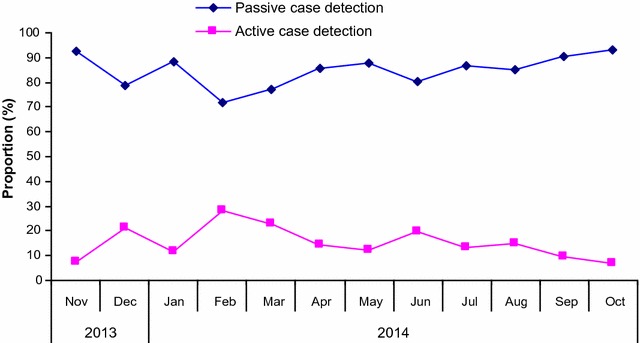
Proportion of fever cases recorded by village health workers during active and passive case detection

During the 12 months of follow-up, 1907 cases with history of fever/and or axillary temperature (≥37.5 °C) were recorded by VHWs through both ACD and PCD. The majority (84.7%) reported through PCD as shown in Fig. 6, out of which 1828 cases (95.9%) were captured using mobile phones and the rest were recorded on paper. About 1778/1907 (93.2%) of cases observed and recorded through either ACD or PCD reported and were registered at the dispensary. However, 44.3% (n = 291) of cases reported through ACD during home visits by VHWs did not report to the dispensary for diagnosis and treatment. Among the patients who did not turn up, 139 (48.8%) had both history of fever at presentation. In terms of age, 58 (19.0%) were under-fives, 55 (18.9%) were aged between 5 and 10 years, 71 (24.4%) were 10–18 years, 107 (36.8%) were aged ≥19 years. There was no association with age (χ^2^ = 4.7, p < 0.192). A higher proportion (55.0%) of females attended than males, and children age under 5 years accounted for 17.4% of all cases (Table 2). Overall, 585 (32.9%) cases attended had fever at presentation (axillary temperature ≥37.5 °C). The majority of fevers came from Magoda compared to other villages (χ^2^ = 22.7, p < 0.001). A higher proportion of fevers were recorded among males compared to females (χ^2^ = 5.2, p = 0.023) and in children aged <1 year (p < 0.001). Some 48.4 and 44.9% of the cases were positive for malaria by RDT and microscopy, respectively, with a significantly higher malaria positivity rate observed at Magoda and Mpapayu villages compared to Mamboleo (p < 0.02). Males had higher malaria parasite positivity rate than females (χ^2^ = 4.6, p = 0.032). The highest malaria parasite positivity rates were detected in children aged 5–9 years (p < 0.001) (Table 2). About 55.0% of fever cases had microscopic malaria parasitaemia and the difference between villages was not statistically significant (p > 0.05) (Table 2). Multivariable logistic regression showed that after adjusting for gender and village, the risk of malaria infection was six times higher in children aged 5–9 years compared to adults aged ≥20 years (AOR = 6.2, 95% CI = 4.3–9.1, p < 0.001), followed by children aged 9–14 years (AOR = 4.2, 95% CI = 2.9–6.3, p < 0.001) and 1–4 years (OR = 3.6 (2.3–5.5, p < 0.01) (Table 3). Furthermore, the risk of malaria was 60% higher in Magoda compared to Mamboleo (AOR = 1.5, 95% CI = 1.1–2.1, p = 0.006) (Table 3).

**Table 2:** Proportion of fevers and malaria cases in the three study villages

Variable	Number attended N (%)	Fever^a^ (≥37.5 °C) n (%)	Malaria by RDT n (%)	Malaria by microscopy n (%)	Cases of fever with malaria^b, c^ n (%)
Villages					
Mamboleo	526 (29.6)	151 (28.7)	217 (41.3)	202 (38.4)	78 (51.7)
Magoda	626 (35.2)	251 (40.1)	324 (51.8)	299 (47.8)	135 (53.8)
Mpapayu	626 (35.2)	183 (29.2)	319 (51.0)	297 (47.4)	107 (58.5)
Test statistics, χ^2^ (p value)		22.7 (p < 0.001)	15.3 (p < 0.001)	12.7 (0.02)	1.7 (0.428)
Gender					
Male	779 (45.0)	283 (35.4)	409 (51.2)	377 (47.2)	178 (61.5)
Female	979 (55.0)	297 (30.3)	451 (46.1)	421 (43.0)	179 (60.3)
Test statistics, χ^2^ (p value)		5.2 (0.023)	4.6 (0.032)	3.1 (0.07)	0.08 (0.76)
Age group					
<1	46 (2.6)	32 (69.6)	5 (10.9)	5 (10.9)	4 (12.5)
1–4	263 (14.8)	124 (47.2)	102 (38.8)	81 (30.8)	51 (40.5)
5–9	409 (23.0)	180 (44.1)	265 (64.8)	240 (58.7)	118 (68.2)
10–14	355 (20.0)	127 (35.8)	217 (61.1)	208 (58.6)	87 (68.0)
15–19	119 (6.7)	28 (23.5)	71 (59.7)	68 (57.1)	23 (82.1)
20+	586 (33.0)	89 (15.2)	200 (34.0)	196 (33.5)	37 (41.1)
Test statistics, χ^2^ (p value)		165 (<0.001)	156 (<0.001)	139.2 (<0.001)	66.5 (<0.001)

^a^Fever at presentation (axillary temperature 37.5 °C)

^b^Denominator were cases with fever at presentation

^c^Malaria parasites detected by microscopy

**Table 3:** Multivariable logistic regression of risk factors associated with malaria among individuals of different age groups in the three villages

Variable	Unadjusted OR (95% CI)	p value	Adjusted OR (95% CI)	p value
Age				
<1	1.2 (0.4–3.5)	0.736	1.1 (0.4–3.2)	0.858
1–4	3.7 (2.4–5.7)	<0.001	3.6 (2.3–5.5)	<0.001
5–9	6.3 (4.4–9.2)	<0.001	6.2 (4.3–9.1)	<0.001
10–14	4.3 (2.9–6.3)	<0.001	4.2 (2.9–6.3)	<0.001
15–19	2.5 (1.4–4.5)	0.001	2.5 (1.5–4.3)	0.001
20+	Reference		Reference	
Gender				
Female	Reference		Reference	
Male	1.3 (1.0–1.6)	0.058	1.0 (0.8–1.3)	0.766
Village				
Mamboleo	Reference		Reference	
Magoda	1.6 (1.2–2.1)	0.001	1.5 (1.1–2.1)	0.006
Mpapayu	1.0 (0.7–1.4)	0.949	0.9 (0.7–1.3)	0.844

*OR* odds ratio, *CI* confidence intervals

**Fig. 7:**
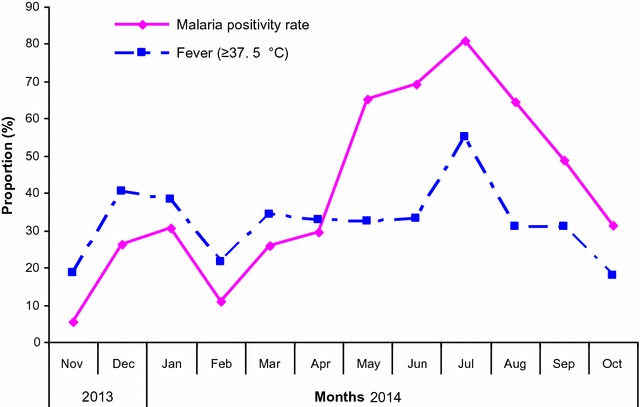
Proportion of fever and monthly malaria cases detected by microscopy

For the entire study period (November 2013 to October 2014), the trends of fever and malaria cases showed bimodal peaks corresponding to the short rain (November/December) and the long rain (April to July) seasons (Fig. 7). The proportion of fevers and malaria cases detected through both ACD and PCD by VHWs is shown in Fig. 6. A slightly higher proportions of cases were recorded in February compared to other months.

### Recurrent fevers and malaria parasite infections

Out of 1778 cases registered at the dispensary, the majority (57.0%) attended once, whereas only 8.3% were seen between four and ten times (Table 4). The proportion of cases with malaria parasite infections increased with the number of visits (χ^2^ = 22.9, p < 0.001). The risk of malaria was two times higher among individuals who visited the dispensary between four and ten times compared to those with a single visit (crude OR = 2.0, 95% CI = 1.4–2.8). However, the proportion of cases with fever did not differ significantly with the number of visits (χ^2^ = 4.4, p = 0.218). The median duration of fever episodes was 59 days (IQR, 30–112 days) and the median duration of malaria episodes by RDTs was 64 days (IQR, 37–116 days).

**Table 4:** Proportion of microscopic malaria cases and fever episodes by number of visits

Visits	Number of cases attended	Malaria cases*		Fever^†^	
		n (%)	OR (95% CI)	n (%)	OR (95% CI)
1	1012	410 (40.5)		325 (32.1)	–
2	435	204 (46.5)	1.3 (1.0–1.6)	150 (34.5)	1.1 (0.9–1.4)
3	183	93 (50.8)	1.5 (1.1–2.1)	66 (36.1)	1.2 (0.9–1. 7)
4–10	148	91 (61.5)	2.3 (1.6–2.3)	39 (26.4)	0.8 (0.5–1.1)
Overall	1778	798 (44.9)		580 (32.6)	

Fever: axillary temperature ≥37.5 °C

*OR* odds ratio, *CI* confidence intervals

* Test statistics (χ^2^ = 27.0, p < 0.001)

^†^ χ^2^ = , 4.4, p = 0.218

### Adherence to treatment and follow-up of patients taking anti-malarials

All patients treated with AL (n = 860) were given the first dose under the supervision of a nurse at the dispensary, whereas 98.2% of the patients took the remaining doses under the supervision of the VHWs. Among the patients treated, 477 (55.5%) were treated only once, 220 (25.6%) were treated twice and 163 (19.0%) were treated three times or more during the entire study period. Those who did not complete full dose could not be assessed during the follow-up on day 7 because they migrated out of the study area. Only nine patients (1.04%) had malaria parasites detected by microscopy on day 7 and were re-treated with AL.

### Incidence of malaria

The incidence of malaria by gender and age category is presented in Table 5. The mean annual incidence of malaria was 281 per 1000 person years (pys) (95% CI = 262–302) and there were no significant difference between females and males (IRR = 1.09, 95% CI = 0. 94–1.25). The incidence rate increased significantly with age and peaked at age 5–9 years (p < 0.001). A similar and high incidence of malaria was observed among individuals aged 5–9 and 10–14 years (p < 0.001), when compared to individuals aged ≥20 years (Table 5).

**Table 5:** Distribution of incidence of malaria by gender and age

Variable	Person-years	Malaria cases	Incidence rate	IRR (CI 95%)	p value
Gender					
Female	1412	415	293.9	1.09 (0.94–1.25)	0.255
Male	1388	374	269	Reference	
Age (years)					
<1	33	5	152	1.06 (0.44–2.59)	<0.001
1–4	285	80	280	1.97 (1.52–2.56)	<0.001
5–9	448	236	527	3.70 (3.06–4.45)	<0.001
10–14	400	205	511	3.59 (3.00–4.36)	<0.001
15–19	258	67	259	1.82 (1.38–2.40)	<0.001
≥20	1376	196	142	Reference	–
Total	2800	789	282	–	–

*IRR* incidence rate ratio

## Discussion

Utilization of mobile phone technology in the developing world has grown rapidly in recent years with a concomitant increase in the coverage and decrease of the price of connectivity. The health sector is tapping into the potential of mobile phone technology to increase efficiency and performance for service delivery. Mobile phone applications have been developed to improve service delivery strategies, disease surveillance, supply chain management of essential medical commodities as well as assisting policy makers in planning and implementing different programmes [13]. In Kenya, the use of *SMS for life* has revealed that mobile phone text messages can be used to transmit timely surveillance data from peripheral health facilities to higher levels [13]. Another study conducted in Zanzibar utilized mobile phones in surveillance of malaria and revealed that mobile phones are among the tools that can help accelerate malaria elimination by improving coordination, timing, coverage, and responses [14].

This was a pilot study that aimed at developing, testing and deploying a mobile phone-based platform and assessing its potential use in the surveillance of febrile illness by VHWs with moderate training. The system was used to capture information on cases with severe febrile illnesses and supporting prompt malaria case management. In addition, it was used to facilitate monitoring of treatment outcomes in areas with declining burden of malaria transmission in recent years [6, 15, 16]. The ComDSTM system was developed through a consultative process between researchers (from NIMR and University of Notre Dame) and an IT team from a private company in India. The process enabled development of different applications that incorporated a logical framework to support the flow of information and cases from the community to the dispensary and within the different steps of case management performed at the dispensary. However, due to limited connectivity and internet requirement of the system, changes were made to allow data capture in areas without internet and for uploading data when connectivity resumed, and/or use of paper-based questionnaires when the system could not function. The system was not locked to any mobile telephone service provider and this allowed VHWs to subscribe to the provider with the best connectivity in their areas. This was critical and will potentially allow future scaling-up of the system and its application in any area with access to mobile phone and data transfer services regardless of the provider.

Optimization and deployment of the ComDSTM system were done in phases that involved theoretical and practical training, and a ‘dry run’ to allow VHWs and dispensary staff to learn and eventually apply the system. Despite the challenges encountered at the beginning, which were mainly associated with limited internet connectivity, the system was effectively deployed and used by all stakeholders. Of special interest and for future applicability, this system can be successfully used by VHWs with minimum training in PCD and ACD of fevers as part of surveillance of malaria in rural communities. This suggests that if the system can be adopted and scaled-up, it might be used by health workers with minimum training in real-time recording and transmission of medical information to a higher level to support decision making and improve case management. However, for future applicability of the ComDSTM at wider geographical coverage in areas with weak internet connectivity, some modifications will be required to allow real-time capture of data without a need for the system to run parallel with paper-based data collection tools. There were some delays experienced in the transfer of information to subsequent steps for case management caused by the absence of internet. A rational solution could possibly involve sending short text messages to the central server, which can automatically relay the required information to other stakeholders and store the data into the central database.

Following the successful deployment of the mobile phone-based system in collecting actively and passively malaria febrile cases data from the community and Magoda dispensary, it enabled the VHWs to monitor the treatment of malaria cases using AL and treatment outcome. However, it was shown that the majority of fever cases were reported through PCD and a large proportion of cases (44.3%) detected through ACD by VHWs and who were referred to the dispensary did not show up. Failure to report to the dispensary by the cases identified at household level could be attributed to the attitude, knowledge, and practices of the community members towards febrile illnesses. Healthcare seeking is not a priority and some community members opt for self-treatment with antipyretics when they have a mild condition, and seek medical care from the health facility when they become severely ill. As shown by previous studies, most of the people in poor countries do not take action against mild illnesses and will seek health services when they get too ill to continue with their socio-economic activities [17]. More studies are needed to explore the factors which might be associated with refusal or delays to report to the dispensary after a referral by VHWs during ACD.

Early detection and treatment of malaria reduces the reservoir of the parasites in the population, as well as transmission of malaria, morbidity and related mortality [18]. This study showed that the majority of cases (≈85%) self-reported to the dispensary and were captured through PCD. Apart from the WHO recommendations to consider active case surveillance in areas with low malaria transmission [19], these findings suggest that ACD might not be suitable and cost-effective for surveillance of symptomatic cases in the study villages and possibly other areas with similar malaria transmission intensity. In the current study, two VHWs were deployed in each of the three study villages while only two VHWs supported the dispensary staff to attend to patients reporting to the dispensary through PCD. Comparatively, the ACD system (with six VHWs) who visited each of the households weekly, was more expensive to run compared to PCD and it captured fewer patients and some that could not report to the dispensary. The population in the study villages has been involved in different studies from 1992 and the villages have readily available services at Magoda dispensary [15]. All cases reported through ACD have access to the health services before they become severely ill. Due to long exposure to research and associated medical care, the community may be considered to have better knowledge of malaria and improved health-seeking behaviour. These findings suggest that with better health services and improved behavioural change communication messages about malaria and care-seeking practices, PCD might be the most appropriate and cost-effective option for surveillance of symptomatic malaria in the study villages and other areas with similar levels of malaria transmission. However, surveillance of asymptomatic carriers who are considered reservoirs and play an important role in malaria transmission, especially in hypo-endemic areas, might not be adequately undertaken by PCD only and this requires further investigation.

The results further revealed that the burden of malaria among infants and under-fives was lower compared to other age groups. This could be due to acquired maternal protection which has been shown to decline with age [20, 21]. In contrast, the burden of malaria was significantly higher among children aged 5–10 and 10–19 years, as shown by recent studies from Northeastern Tanzania where a shift in the malaria burden from young to older children between the age of 10 and 19 years suggested that the area had become hypo-endemic to malaria [6, 15, 16]. The pattern of malaria burden observed in the current study is similar to the situation reported during the recent decline of malaria burden, whereby the prevalence and incidence of malaria usually occurred in children over 10 years old [6, 15, 16], unlike that previously reported in young children where due to high malaria transmission, early exposure to multiple parasite clones was attributed to early development of anti-malarial immunity in high transmission areas, making young children more protected against clinical malaria even when carrying parasites [22]. The sudden increase in malaria burden in the current study could be attributed to the heavy rains which were experienced in 2013 and 2014, leading to an increase in malaria cases, as reported by Shayo et al. [23]. Further studies and sustained surveillance is urgently required to monitor the trends of malaria in areas where the burden of the disease has recently declined but is still resilient to weather changes.

### Study limitations and challenge

The main challenge was difficulties in using the mobile system when the mobile network connectivity or the server was down. This was resolved by documenting the information on paper-based tools and posting data on the relevant platforms whenever connectivity resumed. A similar system was used in some parts of the villages that had relatively poor network connectivity. Furthermore, the data transfer services provided by most of the mobile phone companies in the study area was based on 2G which in most cases was the major limitation for efficient functioning of the ComDSTM system. Another limitation was the absence of alert message components. The system lacked the functionality to remind VHWs to administer drugs to patients, a function which would have helped to ensure drugs were administered in timely fashion.

## Conclusion

The study showed that the ComDSTM system using mobile phone-based technology could be successfully developed, deployed and used by VHWs in surveillance and timely reporting of fever episodes. The system was utilized in the monitoring of treatment failure in remote areas but with access to different types of mobile-based data transfer services. Furthermore, the study showed that PCD was able to capture the majority of cases and yet it was relatively inexpensive compared to ACD, suggesting that in areas of similar malaria transmission, pro-active surveillance may not be the best option for capturing and monitoring symptomatic malaria cases. Optimization and scaling-up of the mobile phone-based ComDSTM system will be required to utilize the tools for improved surveillance of both symptomatic and asymptomatic malaria cases and detection of treatment failures/drug resistance.
